# Evaluation of the safety of high-frequency chest wall oscillation (HFCWO) therapy in blunt thoracic trauma patients

**DOI:** 10.1186/1752-2897-2-8

**Published:** 2008-10-06

**Authors:** Casandra A Anderson, Cassandra A Palmer, Arthur L Ney, Brian Becker, Steven D Schaffel, Robert R Quickel

**Affiliations:** 1 Surgery Department, Hennepin County Medical Center, Minneapolis MN, USA; 2 Clinical Research, Hill-Rom, St Paul MN, USA

## Abstract

**Background:**

Airway clearance is frequently needed by patients suffering from blunt chest wall trauma. High Frequency Chest Wall Oscillation (HFCWO) has been shown to be effective in helping to clear secretions from the lungs of patients with cystic fibrosis, bronchiectasis, asthma, primary ciliary dyskinesia, emphysema, COPD, and many others. Chest wall trauma patients are at increased risk for development of pulmonary complications related to airway clearance. These patients frequently have chest tubes, drains, catheters, etc. which could become dislodged during HFCWO. This prospective observational study was conducted to determine if HFCWO treatment, as provided by The Vest™ Airway Clearance System (Hill-Rom, Saint Paul, MN), was safe and well tolerated by these patients.

**Methods:**

Twenty-five blunt thoracic trauma patients were entered into the study. These patients were consented. Each patient was prescribed 2, 15 minute HFCWO treatments per day using The Vest^® ^Airway Clearance System (Hill-Rom, Inc., St Paul, MN). The Vest^® ^system was set to a frequency of 10–12 Hz and a pressure of 2–3 (arbitrary unit). Physiological parameters were measured before, during, and after treatment. Patients were free to refuse or terminate a treatment early for any reason.

**Results:**

No chest tubes, lines, drains or catheters were dislodged as a result of treatment. One patient with flail chest had a chest tube placed after one treatment due to increasing serous effusion. No treatments were missed and continued without further incident. Post treatment survey showed 76% experienced mild or no pain and more productive cough. Thirty days after discharge there were no deaths or hospital re-admissions.

**Conclusion:**

This study suggests that HFCWO treatment is safe for trauma patients with lung and chest wall injuries. These findings support further work to demonstrate the airway clearance benefits of HFCWO treatment.

## Background

Blunt thoracic trauma can result in a variety of bony and non-bony injuries [1]. These patients are often cared for in the intensive care unit (ICU), and frequently require some form of pulmonary support. Mechanical ventilation carries with it risk for additional complications such as atelectasis and ventilator associated pneumonia (VAP). Patients requiring intubation often require longer ICU stays [2]. Avoiding mechanical ventilatory support of patients who don't absolutely require it results in a better outcome for these patients [3]. Blunt thoracic trauma patients and patients with flail chest have been treated effectively with bilevel positive pressure (BiPAP), continuous positive airway pressure (CPAP), or intermittent positive pressure ventilation (IPPV), with improved outcomes resulting from BiPAP and CPAP [2,4]. A recent study of mucociliary clearance in ICU patients demonstrated a significant deficit in clearance ability that correlated to patient acuity [5]. Effective mucociliary clearance is an essential first line of defence to maintaining respiratory health [6] and impairment of this clearance may contribute to the risk for pulmonary complications during an ICU stay.

There are different methods which have been employed to facilitate pulmonary clearance. Conventional chest pulmonary therapy (CPT), which consists of manual percussion and or positioning techniques (postural drainage) to help mobilize and clear mucus is one. Continuous Lateral Rotational Therapy (CLRT), which consists of alternatively elevating one lung over the other around the patient's long axis has been shown to help mucus transport [7].

**Figure 1:**
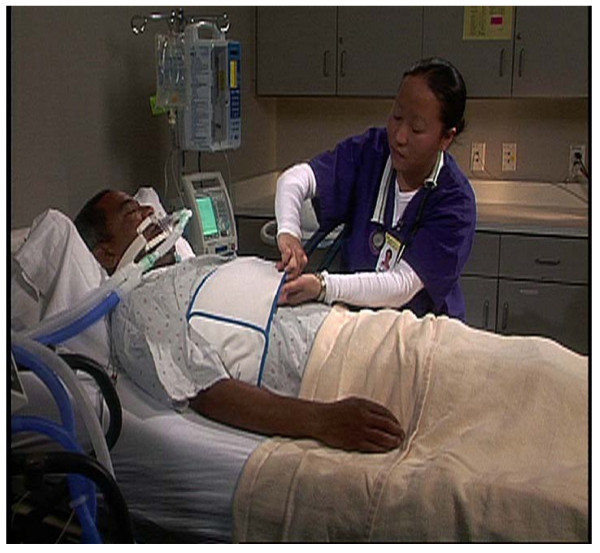
**Intubated patient being fitted for HFCWO treatment**. The vest type being fitted is the "wrap type" of vest. This allows for positioning of the vest so it does not interfere with chest tubes or lines.

High frequency chest wall oscillation (HFCWO) uses a pressurized vest to transmit high frequency oscillations to the chest. This mobilizes secretions which are then cleared by cough or by suction in the case of intubated patients. Early trials of HFCWO demonstrated mucus clearance in dogs [8]. Studies in humans found that HFCWO helps tracheal mucus clearance [9]. This clearance decreases pulmonary complications in patients with chronic pulmonary diseases [10,11]. A comparison of clearance in hospitalized patients demonstrated equivalent efficacy of CPT and HFCWO [12,13]. Figure 1 shows an intubated patient being prepared for HFCWO treatment.

Since it was shown that airway clearance would be advantageous for thoracic trauma patients, HFCWO had been shown to be equivalent to CPT in patients with other clearance needs, and CPT was tolerated by blunt thoracic trauma patients, we hypothesized that:

• Treatment with HFCWO therapy will result in no significant changes in physiological parameters in patients with chest wall injuries (CWI).

• There will be no increase in the number of adverse outcomes related to treatment with HFCWO in patients with CWI.

## Methods

**Figure 2:**
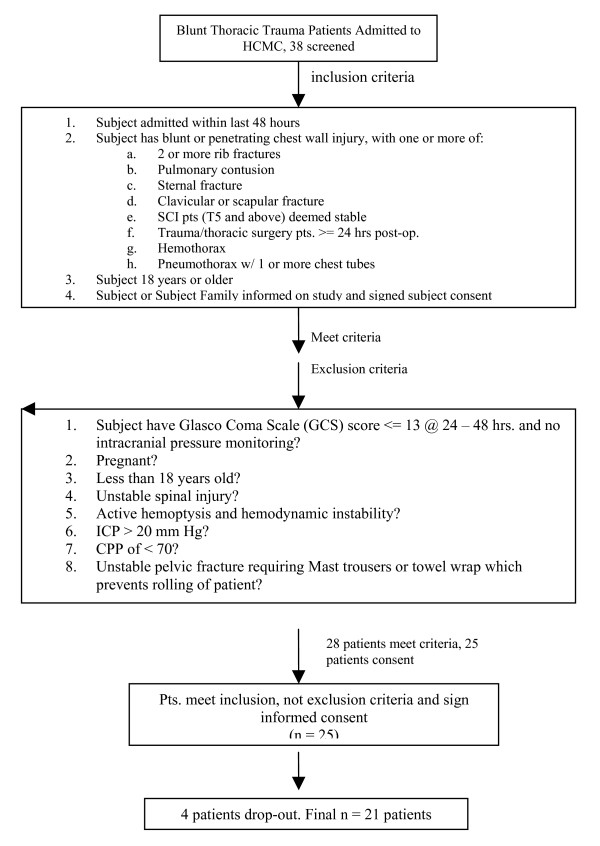
**Flow Chart showing patient selection and exclusion**. A total of 38 patients were screened. Of these, 28 patients met criteria. Twenty-five patients consented but four did not complete the therapy course. Twenty one patients were evaluated and followed-up for evaluation of HFCWO treatment. As an observational pilot safety study, power calculations for the number of patients to be enrolled were not done. The target of 25 patients was arbitrary.

This study was approved by the institution's Institutional Review Board. Patients (18 years or older) with chest wall injury (blunt or penetrating) admitted to the Hennepin County Medical Center trauma service (Minneapolis, Minnesota) with one or more of the following: a) Two or more rib fractures (unilateral or bilateral); b) Pulmonary contusion as the result of direct force applied to the lung documented as an area of increased density or consolidation by chest x-ray; c) Sternal fracture; d) Clavicular or scapular fracture; e) Spinal cord injury patients (T5 and above) deemed stable by the neurological staff; f) Hemothorax; or g) Pneumothorax requiring one or more chest tubes were recruited into the study after informed consent. These criteria were documented with chest radiograph (CXR), or by chest or neck computerized tomography (CT) scan. In addition, patients had to have been admitted within the previous 48 hours. See figure 2 for a flow diagram.

Since this was a safety study and not a controlled comparison, power calculations were not performed. The selection of 25 patients was empirical. Only the actual number of patients enrolled was recorded. The total number screened was not documented.

The first twenty-five eligible patients to consent were enrolled in the study over approximately 10 months. Each patient was prescribed 2, 15 minute HFCWO treatments per day using The Vest^® ^Airway Clearance System (Hill-Rom, Inc., St Paul, MN). The Vest^® ^system was set to a frequency of 10–12 Hz and a pressure of 2–3 (arbitrary unit). Physiological parameters were measured before, during, and after treatment. These parameters were: incentive spirometry; heart rate; heart rhythm; respiratory rate; mean arterial pressure calculated from systolic and diastolic blood pressure; O_2 _saturation (SpO_2_); and in selected cases, intra-cranial pressure. For those patients receiving mechanical ventilation, the following ventilator settings were documented: ventilation mode; ventilation rate; PEEP; pressure support level; delivered tidal volume; FiO_2_; peak pressure. All treatments were offered to the patients, but some were skipped according to the patient's wishes (16 total treatments skipped due to pain, 7 treatments due to nausea) or early discharge (25 treatments). Because of inconsistencies in recording the "during treatment" parameters, only the before and after treatment parameters were compared. Student's *t*-test for paired values was used and values were considered significant at a p value of 0.05. All patients were followed-up after 30 days to determine: subject health status, number of hospital days required, number of ICU days required, number of days on mechanical ventilation (if applicable), incidence of re-intubation (if applicable), hospital re-admissions for pulmonary complications, and subject assessment of HFCWO therapy (comfort/tolerance).

## Results

Twenty-five patients were initially enrolled. Four withdrew prior to the completion of the study. None withdrew as a result of adverse effects of the HFCWO treatment. Table 1 presents the age and sex of the patients who remained in the study, the type of trauma injuries and the adjunct equipment at the time of HFCWO treatment. None of the equipment was dislodged or compromised in function by the HFCWO treatment. The majority of trauma was the result of motor vehicle accidents but also included 3 falls, 1 industrial accident, and 1 gunshot wound to the chest.

**Table 1:** Patient demographics, diagnoses, and adjunctive equipment present during HFCWO therapy

Patient #	Age	Gender	Injuries	Adjunct equipment
01	39	M	rib fract, scapular fract, pneumothorax, h	None
02	68	M	rib fract, clavicle fract, stab spinal cord fract, pneumothorax	chest tube
04	40	F	pulm contusion	other line
05	48	M	rib fract	other line
06	18	F	rib fract, pulm contusion, clavicle fract, scapular fract, pneumothorax	other line
07	19	M	rib fract, pulm contusion, clavicle fract, pneumothorax	chest tube, DS, other line
09	62	M	pneumothorax	chest tube, other line
10	44	F	rib fract	other line
11	26	M	rib fract, pneumothorax	chest tube, other line
12	39	M	rib fract, sternal fracture, pneumothorax	chest tube, other line
13	68	M	rib fract, liver laceration, kidney laceration, pneumothorax	other line
14	70	M	rib fract, pneumothorax	chest tube, other line
15	44	F	rib fract, pulm contusion, pneumothorax	other line
16	49	F	rib fract, pulm contusion, pneumothorax	chest tube, other line
19	44	M	rib fract, pulm contusion	other line
20	21	M	rib fract, clavicle fract, stab spinal cord fract	other line
21	59	M	rib fract, pneumothorax, intraperitoneal hemorrhage	other line
22	42	F	rib fract, pulm contusion, hemothorax, pneumothorax	other line
23	51	M	pulm contusion, hemothorax	chest tube, other line
24	70	M	rib fract, pulm contusion, pneumothorax	chest tube, other line
25	44	F	rib fract, hemothorax, pneumothorax, kidney laceration	chest tube, other line

Seven patients initially admitted with small pneumothoraces were treated conservatively; none of these patients required tube thoracostomy after HFCWO. In patients with chest tubes (n = 11), mean chest tube output during treatment and the 30 minutes following was 10 cc (range 0–50 cc). One patient with flail chest and a large pleural effusion required chest tube placement after the first HFCWO treatment due to increasing serous pleural fluid; treatments were continued without further incident. None of the ten patients with solid organ injury being managed non-operatively required transfusion or operative management. Use of HFCWO did not result in increased bleeding or need for surgical treatment of solid organ injury in those subjects that were not scheduled for surgery.

Table 2 presents the results of physiologic parameters measured before and after HFCWO treatment. Heart rhythm is not included as there were no remarkable changes pre and post treatment. Mean arterial pressure was measured for some time points for some of the patients (data not shown). None were significantly different than the pre- or post-treatment values. The mean number of treatments each patient received was 7.7.

**Table 2:** Physiological parameters measured before and after HFCWO (Vest) treatment

Parameter	Before Tx Mean (SD) (95% CI)	After Tx Mean (SD) (95% CI)	P value
Incentive Spirometry (cc)	1330.8 (582.0) (166.8 – 2502.8)	1349.8 (544.7) (260.4 – 2439.2)	0.81
Respiratory Rate (bpm)	18.1 (4.0) (10.1 – 26.1)	18.1 (3.9) (10.3 – 25.9)	0.95
Heart Rate (bpm)	91.4 (16.6) (58.2 – 124.6)	91.0 (15.9) (59.2 – 122.8)	0.80
Systolic BP (mm Hg)	131.1 (16.9) (97.3 – 164.9)	128.9 (16.7) (95.5 – 162.3)	0.24
Diastolic BP (mm Hg)	70.1 (12.5) (45.1 – 95.1)	68.3 (12.9) (42.4 – 94.1)	0.21
MAP	90.4 (12.5) (65.4 – 115.4)	88.6 (12.7) (63.2 – 114)	0.20
SaO2 (%)	95.9 (2.7) (90.5 – 101.3)	95.9 (2.7) 90.5 – 101.3)	0.85

The 30 day follow-up survey revealed no deaths or hospital re-admissions. Two patients required re-intubation. One of them was diagnosed with pneumonia. Patient 24 was intubated and heavily sedated during the HFCWO treatment and did not remember the therapy. The other patients were asked about their experience with the Vest. Their responses are shown in Table 3. Seventy-five percent experienced mild or no pain due to the Vest therapy itself. Seventy percent felt the therapy made their breathing better. Seventy-five percent felt the treatment improved their cough and seventy percent would recommend this therapy.

**Table 3:** Patient survey results

	Describe the pain you experienced during Vest therapy				How do you feel overall this therapy made your breathing?			Did the vest therapy improve your cough?			Would you recommend this therapy?		
ID	None	Mild	Moderate	Severe	Better	Worse	No Change	Yes	No	No Change	Yes	No	Unsure

0001		X			X			X			X		
0002			X		X				X		X		
0004		X					X	X				X	
0005		X			X			X			X		
0006	X				X			X			X		
0007			X		X			X					X
0009		X					X		X				X
0010		X			X			X			X		
0011			X				X	X			X		
0012		X			X			X			X		
0013	X				X			X			X		
0014		X			X			X			X		
0015		X					X			X			X
0016			X				X		X			X	
0019		X			X			X			X		
0021		X			X			X			X		
0022	X						X	X					X
0023	X				X			X			X		
0025			X		X			X			X		
Sum	4	10	5		14	0	6	15	3	2	14	2	4
%	20	50	25	0	70	0	30	75	15	10	70	10	20

## Discussion

This study was undertaken to see if HFCWO would be safe and tolerated by patients with blunt chest wall trauma. Studies have shown that HFCWO can aid in the process of airway clearance for hospitalized patients with or without ventilator support [12,13]. The patients in this study tolerated the therapy well and typically did not require additional medication for pain management, despite the severity of their injuries. There were no lines, chest tubes, drains or epidural/ventriculostomy catheters dislodged.

Maintaining pulmonary function in the compromised critically ill patient is challenging. Patients with thoracic trauma are compromised mechanically so that ancillary methods such as mechanical ventilation are often required. However, numerous studies have shown that ventilator-associated pneumonia (VAP) rates can be as high as 65% [14]. Pulmonary clearance is important to prevent VAP, but critical illness impairs the function of the normal mechanisms [5]. Bacterial infections both disrupt the ciliary beat frequency [15] and induce the release of inflammatory components which in turn causes mucus production [16-20]. It is well documented that HFCWO is efficacious for pulmonary clearance in CF patients [21,22] so it is reasonable to try HFCWO for clearance in other conditions.

The overall care of the thoracic trauma ICU patient may be improved by the addition of airway clearance modalities. However, the safety of the device when used by the blunt trauma patient had not been previously demonstrated. The thirty day follow up was an arbitrary time. The intention was to query the patients after significant healing in order to get a more objective opinion of their response to the therapy.

One of the limitations of the study is the lack of documentation of the conditions of the patients who did not participate. It is possible that they represented an overall more seriously injured group which would not find the treatment as tolerable as the included patients. Another limitation is the lack of randomization to conventional CPT or HFCWO therapy. It is not known if the results would be weighted toward one method or the other. However, we felt it was crucial to know that the Vest treatment would be tolerated and as safe as CPT.

The results presented here demonstrate that HFCWO therapy is well tolerated by patients with blunt thoracic trauma and support additional studies to see if the airway clearance capabilities of HFCWO add to the successful treatment of thoracic trauma patients. If so, this method will add another tool to free-up the provider of manual CPT for other patients. It will also add a consistent, technique independent therapy which can be modified (pressure, frequency, and duration) to best accommodate a patients needs.

## Conclusion

This study suggests that HFCWO treatment is safe for trauma patients with lung and chest wall injuries. These findings support further work to demonstrate the airway clearance benefits of HFCWO treatment.

## Abbreviations

BiPAP: bilevel positive airway pressure; CPAP: continuous positive airway pressure; CPT: chest pulmonary therapy; IPPV: intermittent positive pressure ventilation; HFCWO: high frequency chest wall oscillation; VAP: ventilator associated pneumonia; PEEP: positive end expiratory pressure.

## Competing interests

Funding for this study was provided by Hill-Rom, Inc. Brian Becker and Steven D Schaffel are employees of Hill-Rom. Casandra A Anderson, Cassandra A Palmer, Arthur L Ney, Robert R Quickel declare they have no competing interests.

## Authors' contributions

CAA selected and enrolled patients, gathered and interpreted data and summarized results. CAP enrolled and evaluated patients, reviewed results. ALN managed ICU and trauma physicians, evaluated data, assisted on manuscript. BB designed and managed study, data collection forms, provided data analysis. SDS wrote the manuscript, evaluated data, reviewed patients' charts. RRQ admitted patients, monitored clinical condition, prescribed HFCWO treatments and medication. All authors read and approved the final manuscript.

## References

[B1] Schorr RM, Crittenden M, Indeck M, Hartunian S, Rodriguez A (1987). Blunt thoracic trauma, analysis of 515 patients. Ann Surg.

[B2] Xirouchaki N, Kondoudaki E (2005). Noninvasive bilevel positive pressure ventilation in patients with blunt thoracic trauma. Respiration.

[B3] Richardson JD, Adams L, Flint LM (1982). Selective management of flail chest and pulmonary contusion. Ann Surg.

[B4] Gunduz M, Unlugenc H, Ozalevli M, Inanoglu K, Akman H (2005). A comparative study of continuous positive airway pressure (CPAP) and intermittent positive pressure ventilation (IPPV) in patients with flail chest. Emerg Med J.

[B5] Nakagawa NK, Franchini ML, Driusso P, de Oliveira LR, Saldiva PHN, Lorenzi-Filho G (2005). Mucociliary clearance is impaired in acutely ill patients. CHEST.

[B6] Randell SH, Boucher RC (2006). Effective mucus clearance is essential for respiratory health. Am J Respir Cell Mol Biol.

[B7] Dolovich M, Rushbrook J, Churchill E, Mazza M, Powles AC (1998). Effect of continuous lateral rotational therapy on lung mucus transport in mechanically ventilated patients. J Crit Care.

[B8] King M, Phillips D, Gross D, Vartian V, Chang HK, Zidulka A (1983). Enhanced tracheal mucus clearance with high frequency chest wall compression. Am Rev Respir Dis.

[B9] King M, Phillips D, Zidulka A, Chang HK (1984). Tracheal mucus clearance in high-frequency oscillation: chest wall versus mouth oscillation. Am Rev Respir Dis.

[B10] Chiapetta A, Mendendez A, Gozal D, Kiernan M (1996). High-frequency chest wall oscillation in hospitalized non-cystic fibrosis patients. Am J Respir Crit Care Med.

[B11] Hansen L, Warwick W (1990). High-frequency chest compression system to aid in clearance of mucus from the lung. Biomed Instrum Technol.

[B12] Whitman J, Van Beusekom R, Olson S, Worm M, Indihar F (1993). Preliminary evaluation of high-frequency chest compression for secretion clearance in mechanically ventilated patients. Respir Care.

[B13] Arens R, Gozal D, Omlin KJ, Vega J, Boyd KP, Keens TG, Woo MS (1994). Comparison of high-frequency chest compression and conventional chest physiotherapy in hospitalized patients with cystic fibrosis. Am J Respir Crit Care Med.

[B14] Evans B (2005). VAP prevention. Nursing Management.

[B15] Steinfort C, Wilson R, Mitchell T, Feldman C, Rutman A, Todd H, Sykes D, Walker J, Saunders K, Andrew PW, Boulnois GJ, Cole PJ (1989). Effect of *Streptococcus pneumoniae *on Human Respiratory Epithelium In Vitro. Infect Immun.

[B16] Park JA, He F, Martin LD, Yuehua L, Chorley BN, Adler KB (2005). Human Neutrophil Elastase Induces Hypersecretion of Mucin from Well-Differentiated Human Bronchial Epithelial Cells *in Vitro *via a Protein Kinase Cδ-Mediated Mechanism. American Journal of Pathology.

[B17] Wilson R, Dowling RB, Jackson AD (1996). The biology of bacterial colonization and invasion of the respiratory mucosa. Eur Respir J.

[B18] Dohrman A, Miyata S, Gallup M, Li JD, Coste A, Escudier E, Nadel J, Basbaum C (1998). Mucin gene (MUC 2 and MUC 5AC) upregulation by Gram-positive and Gram-negative bacteria. Biochim Biophys Acta.

[B19] McNamara N, Basbaum C (2001). Signaling networks controlling mucin production in response to Gram-positive and Gram-negative bacteria. Glycoconj J.

[B20] Basbaum C, Li D, Gensch E, Gallup M, Lemjabbar H (2002). Mechanisms by which Gram-positive bacteria and tobacco smoke stimulate mucin induction through the epidermal growth factor receptor (EGFR). Novartis Found Symp.

[B21] Tecklin JS, Clayton RG, Scanlin TF (2000). High frequency chest wall oscillation *vs*. traditional chest physical therapy in CF – a large, one-year, controlled study. Pediatr Pulmonol.

[B22] Darbee JC, Kanga JF, Ohtake PJ (2005). Physiological evidence for high-frequency chest wall oscillation and positive expiratory pressure breathing in hospitalized subjects with cystic fibrosis. Physical Therapy.

